# Influence of Licorice Root Feeding on Chemical-Nutritional Quality of Cow Milk and Stracciata Cheese, an Italian Traditional Fresh Dairy Product

**DOI:** 10.3390/ani9121153

**Published:** 2019-12-16

**Authors:** Francesca Bennato, Andrea Ianni, Denise Innosa, Camillo Martino, Lisa Grotta, Francesco Pomilio, Micaela Verna, Giuseppe Martino

**Affiliations:** 1 Faculty of BioScience and Technology for Food, Agriculture and Environment, University of Teramo, Via Renato Balzarini 1, 64100 Teramo (TE), Italy; francescabennato82@gmail.com (F.B.); dinnosa@unite.it (D.I.); lgrotta@unite.it (L.G.); micaelaverna1997@gmail.com (M.V.); 2 Department of Medical, Oral and Biotechnological Sciences, “G. d’Annunzio” University Chieti-Pescara, Via dei Vestini 31, 66100 Chieti, Italy; andreaianni@hotmail.it; 3 Specialist Diagnostic Department, Istituto Zooprofilattico Sperimentale dell’Abruzzo e del Molise “G. Caporale” Via Campo Boario, 64100 Teramo (TE), Italy; c.martino@izs.it; 4 Food Hygiene Unit, NRL for *L. monocytogenes*, Istituto Zooprofilattico Sperimentale dell’Abruzzo e del Molise “G. Caporale” Via Campo Boario, 64100 Teramo (TE), Italy; f.pomilio@izs.it

**Keywords:** dairy cow, fatty acid, volatile profile, Stracciata cheese

## Abstract

**Simple Summary:**

The aim of this study was to investigate the effects of dietary licorice root supplementation on chemical and nutritional characteristics of cow milk and Stracciata cheese, an Italian traditional fresh dairy product. Our results suggest a positive role of licorice in improving the nutritional and organoleptic properties of dairy cow products, influencing various parameters such as fatty acid and volatile profiles.

**Abstract:**

The aim of this study was to evaluate the effect of dietary licorice root supplementation on chemical and nutritional characteristics of cow milk and Stracciata cheese. Licorice did not influence milk and Stracciata fat content but induced modifications in fatty acid profile. Both in milk and Stracciata, a decrease in saturated fatty acids was detected and in cheeses an increase of monounsaturated and polyunsaturated fatty acids was observed. Stracciata obtained from the experimental group showed an improved oxidative stability after six days of ripening, a finding partly justifiable by the well-known richness of antioxidant compounds in the licorice root. The volatile profile of Stracciata was also affected by dietary licorice intake, with an increase in esters and a reduction of aldehydes and ketones. These results suggest a positive role of licorice in modifying chemical and physical properties of cow cheeses, reducing lipid oxidation and inducing changes in color and flavor with a presumable improvement in consumer acceptability.

## 1. Introduction

Licorice (*Glycyrrhiza glabra L.*) is a perennial plant, widespread in south and central Europe. The licorice roots are harvested and processed for use in the food industry and medicine, and dried leaves can be used as roughage for ruminants in semi-arid and arid regions. The phytochemical composition of licorice is very complex, comprising triterpene saponins, a complex mixture of flavonoids, including glycosides and aglycones with flavonoid, isoflavonoid, and polysaccharides. Some of licorice constituents are metabolized by gut microbiota leading to the formation of metabolites like 18-ß-glycyrrethinic acid [1,2]. This metabolite, an aglycone of the triterpenoid glycyrrhizic acid, is better absorbed into systemic circulation than glycyrrhizic acid and it is known to possess a wide variety of pharmacological effects [3]. The use of licorice root as feed additive has been investigated mainly in monogastrics [4,5] and scarcely in ruminants. However, licorice has received great interest as a natural antioxidant due to its high amount of triterpene, saponins, and flavonoids and several phenolic acids, such as liquiritigenin, liquiritin, isoliquiritigenin, isoliquiritin, glabridin, glabrene, licochalcone, and glycycoumarin. 

Several studies demonstrated that feeding strategies based on the use of plants rich in bioactive compounds are effective in influencing the chemical-nutritional composition profile of milk and derived dairy products [6,7]. To date, only a few studies have reported the effect of dietary licorice supplementation on chemical-nutritional composition of milk and cheese. Zhang et al. (2015) [8] demonstrated that dietary supplementation of 0.4% licorice root extract in sheep resulted in greater antioxidant capacity of the meat, increased antioxidant content and radical scavenging activity and decreased reactive oxygen species (ROS) and thiobarbituric acid reactive substance (TBARS) levels of the meat. Moreover, additional studies conducted on sheep suggested the ability of licorice to modulate rumen fermentation if used as diet supplementation [9]. More recently, it has been demonstrated in goats that a dietary enrichment with fresh licorice roots (1% on dry matter) caused a reduction of somatic cell count (SCC), an increase in the protein component in milk with consequent improvement of technological properties, and an increase of the oxidative stability of ripened cheeses [10]. 

The present work aimed to investigate the effect of dietary licorice root supplementation on chemical-nutritional characteristics, fatty acid (FA) profile, oxidative stability, and volatile compounds (VOCs) of milk and Stracciata. Stracciata is an Italian traditional fresh and soft dairy product made from cow milk. The name *Stracciata* is derived from the verb *stracciare*, meaning *to tear*, an action that is made for the formation of this product. It looks like a wide, white strip of elastic, soft spun paste, without crust, about 50 cm long, folded three times. 

## 2. Materials and Methods

### 2.1. Experimental Design

The present experiment was performed according to Directive 2010/63/EU of the European Parliament (European Union, Brussels, Belgium, 2010) and Directive 86/609/ EEC (European Economic Community, Brussels, Belgium, 1986), which deals with the protection of animals used for scientific purposes [11,12].

This study was conducted for a period of 30 days on sixteen cows, homogeneous for age and lactation period. Animals were randomly divided into two groups of eight cows each: a control group (CG) and an experimental group (EG) whose diet was supplemented with licorice root. During the entire experimental period, CG cows received food in the form of “unifeed” a standard complete food formulated for the nutritional needs of cows in mid-lactation. The EG cows received the same complete food, formulated according to the same requirements and prepared in the same way, but the daily ration of each cow was supplemented with 20 g of licorice root. 

### 2.2. Stracciata Cheese Manufacturing

On day 30, bulk milk was collected separately from each group and used partly for chemical analysis and partly for Stracciata cheesemaking. The adopted manufacturing protocol provided the initial addition of citric acid to raw bovine milk that was subsequently heated until to reach a temperature of 38 °C. After that, a selected lactic culture characterized by omofermentant *Streptococcus thermophilus* (Activo Select Lactic Culture, UniFerm srl, Calitri, Italy) was added and after 30 min there was the rennet (Naturen Premium 225, Chr. Hansen, Italy) addition. Milk coagulated within 40 min at 32–33 °C. Once curdling was completed, the cheese mass was transferred to a table and cut into approximately cube-shaped with sides of 1.5–2.0 cm. After 2 h of draining without pressing, the cheese mass was salted with 5% sodium chloride water solution preventively heated at 85 °C. 

Two different cheesemakings were performed, each of which yielded three Stracciata cheeses for the CG and three Stracciata cheeses for the EG. All cheeses were stored at 4 °C. For each group, three cheeses were sampled after 1 day (T1) from the cheesemaking, while the rest were stored for up to 6 days (T6). T1 samples were analyzed for chemical composition, color, fatty acid profile, volatile profile, and oxidative stability; T6 samples were used only to evaluate changes in volatile profile and oxidative stability.

### 2.3. Chemical Analysis of Milk and Stracciata

Chemical composition of milk (fat, protein, casein, lactose, and urea) was determined using a MilkoScan FT 6000 (Foss Integrator IMT; Foss A/S, Hillerod, Denmark). Stracciata DM content was determined according to AOAC International (2000) [13]. Fat in Stracciata was extracted by acid hydrolysis, by homogenizing 2.5 g of cheese with 20 mL of ethanol and 500 μL of 3 N hydrochloric acid. The amount of fat was expressed as mean percentage on DM.

### 2.4. Colorimetric Analysis of Stracciata

A Chroma Meter CR300 spectrophotometer (Minolta, NY, USA) was used to measure Stracciata color and to calculate L* (lightness), a* (green-red), and b* (blue-yellow) values. Color measurements of each sample were performed on one of the smooth faces of the cheeses previously cut. Furthermore, total color difference (ΔE*ab) and the yellow index (YI) were calculated. Final values of ΔE*ab were compared according to Zmeškal et al. (2002) [14].

### 2.5. Stracciata and Milk FA Profile

Milk FA fraction was extracted according to the official AOAC method [13], while, in the case of Stracciata, the fat was extracted according to the Folch method [15]. 

In both milk and cheese, 50 mg of fat was dissolved with 1 mL of hexane and 500 μL of methylant agent sodium hydroxide (final concentration 2 M) in methanol was added. The milk and relative dairy products profiles were evaluated by a gas chromatography-flame ionization detector (GC-FID) (Focus GC; Thermo Scientific, Walthman, MA, USA) equipped with a capillary column (Restek Rt-2560 Column fused silica 100 m × 0.25 mm highly polar phase; Restek Corporation, U.S., Benner Circle, Bellefonte, PA 16823, USA). Hydrogen was used as carrier gas. The initial holding temperature was 80 °C for 10 min; then it was increased to 172 °C at a rate of 4 °C min^−1^ and held for 30 min. The final temperature of 190 °C was reached at a rate of 4 °C min^−1^ and was held for 10 min. Peak areas were quantified using ChromeCard software and the relative value of each individual FA was expressed as a percentage of the total FA. The value of each FA was used to calculate the sum of monounsaturated fatty acids (MUFA), polyunsaturated fatty acids (PUFA), and saturated fatty acids (SFA). Desaturation indices (ΔI) were calculated using the formula suggested by Brogna et al. (2011) [16].

### 2.6. Evaluation of the Oxidative State and Volatile Compounds in Stracciata

Lipid oxidation in Stracciata after 1 (T1) and 6 (T6) days of storage was determined by the quantification of TBARS according to the procedure reported by Ianni et al. (2019) [17]. Briefly, about 2.5 g of cheese were homogenized with 500 μL of 0.01% butylated hydroxytoluene (BHT) in methanol and 20 mL of trichloroacetic acid (TCA) 75 g L^−1^. The mixture was centrifuged and subjected to distillation. An aliquot of 5 mL of the filtrate was mixed with an equal volume of a 0.02 M thiobarbituric acid (TBA) solution in 90% acetic acid. The solution was heated in a thermostated bath at 95 °C for 45 min and after cooling, the absorbance at 534 nm was evaluated with a JENWAY 6305 UV/vis Spectrophotometer (Barloworld Scientific, Milan, Italy). The results were expressed in μg of malondialdehyde (MDA) g^−1^ of cheese.

The VOCs in T1 and T6 Stracciata were extracted by solid phase micro-extraction (SPME) and analyzed with a gas chromatograph (GC) coupled with a mass spectrometer (MS) (Perin Elmer, Waltham, MA, USA) equipped with an Elite-5MS column (30 × 0.25 mm, 0.25 μm, Phenomenex, Torrance, CA, USA). Three grams of cheese were mixed with 10 mL of saturated NaCl solution (360 g L^−1^) and 10 μL of internal standard solution (4-methyl-2-heptanone; 10 mg kg^−1^ in ethanol). The volatile compounds were extracted from the head space of the vial with a divinylbenzene-carboxen-polydimethylsiloxane SPME fiber (length: 1 cm; film thickness: 50/30 μm; Supelco) directly exposed for 60 min at 60 °C. VOCs were thermally desorbed into the GC injector splitless mode at 250 °C for 30 min. The thermal program and the identification of the VOCs was carried out according to the protocol previously described [18]. 

### 2.7. Statistical Analysis

Experiments were conducted at least in triplicate. The data were analyzed statistically using SigmaPlot 12.0 software (Systat software, Inc., San Jose, CA, USA) for Windows operating system. The data are tabulated as mean ± standard deviation. One way analysis of variance (ANOVA) was performed. Significantly different groups were ranked using the post hoc comparison tests (Tukey test) with confidence levels set at 95% (*p* < 0.05) and 99% (*p* < 0.01). 

## 3. Results

### 3.1. Nutritional Composition of Milk and Stracciata

Dietary licorice integration did not affect milk composition, in fact no significant differences were observed between CG and EG samples, either in terms of chemical quality (fat, proteins, casein, and lactose), or as regards the ureic content (Table 1).

In Table 2 are reported the chemical composition and the color characteristics of Stracciata cheese sampled after 1 day (T1) from the cheesemaking. No significant differences in fat content were observed; the only variation concerned the higher dry matter (DM) in EG samples (*p* < 0.01). 

Statistically significant changes between the two groups were detected in the color parameters of Stracciata. The colorimetric analysis showed modifications for all the parameters (L*, b*, and a*) in relation to diet. A significant increase in L* (*p* < 0.01) and b* (*p* < 0.001) was detected in the EG with respect to the CG. On the contrary, a reduction (*p* < 0.05) of a* parameter was observed. Differences in YI were observed between the two groups with a higher (*p* < 0.001) YI in EG samples. According to the criteria of Zmeškal et al. (2002) [14], a middle color difference (ΔE*ab) was observed for Stracciata between treated and control group. 

### 3.2. Fatty Acid Profile of Milk and Stracciata

The FA composition of milk and Stracciata is reported in Table 3. Bulk milk showed a significant reduction (*p* < 0.05) in SFA in the EG compared to the CG, with a lower stearic acid (C16:0; *p* < 0.01) content in the EG. No significant differences were observed between the two group in MUFA and PUFA, however in EG samples myristoleic acid (C14:1; *p* < 0.001), palmitoleic acid (C16:1; *p* < 0.001), linoleic acid (C18:2; *p* < 0.01), and α-linolenic (C18:3; *p* < 0.01) increased, while a decrease of vaccenic acid (C18:1, t11; *p* < 0.01) and conjugated linoleic acids CLA (*p* < 0.001) was detected. 

Similarly, the variations evidenced in milk were observed in Stracciata. A significant decrease in SFA was observed in EG samples, however, an increase in SFA > 16 carbon atoms was observed. A significant increase was observed in MUFA (*p* < 0.05) and PUFA (*p* < 0.01). In particular, myristoleic acid (C14:1; *p* < 0.05), vaccenic acid (C18:1, t11; *p* < 0.01), oleic acid (C18:1, c9; *p* < 0.05), and linoleic acid (C18:2, *p* < 0.01) increased. Both in milk and corresponding dairy products, the increase of myristoleic acid (C14:1) was associated with an increase in the related ΔI. Furthermore, both in milk and the relative cheese, an increase of ΔI 16:1 and a reduction of ΔI CLA were observed. 

### 3.3. Analysis of Oxidative State and Volatile Compounds in Stracciata

**Figure 1:**
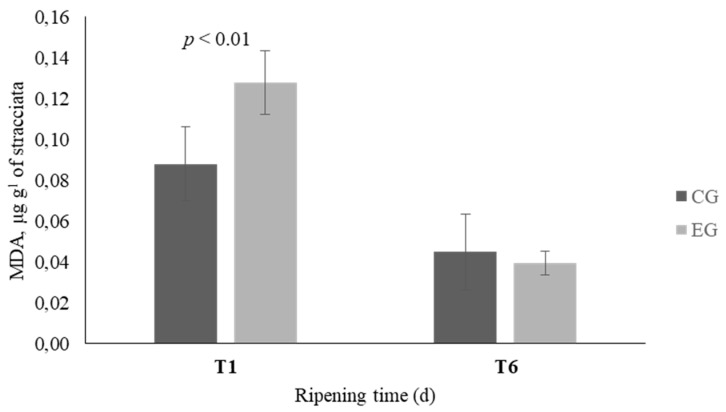
Oxidation profile of Stracciata obtained from the control group (CG—dark columns) and experimental group (EG—light columns). Analysis performed on sample after 1 (**T1**) and 6 (**T6**) d of storage. Data are reported as mean ± standard deviation. MDA = malondialdehyde.

The analysis of the oxidative state of Stracciata at T1 and T6 revealed a different trend between the two groups (Figure 1). At the beginning of the storage, the amount of the oxidation products in the EG samples was higher than the control (*p* < 0.01) and at T6 it was significantly decreased both in the CG and the EG but the level of decrease in the EG was two times more than that of the CG.

Analysis of VOCs was conducted on a sample of Stracciata at T1 and T6 but no significant differences were observed between fresh and stored products. After that time, only results related to T6 samples were reported. In T6 Stracciata samples three families of compounds were detected: esters, aldehydes, and ketones. In Stracciata obtained from cows fed with licorice supplementation the most abundant class was represented by esters. 

**Figure 2:**
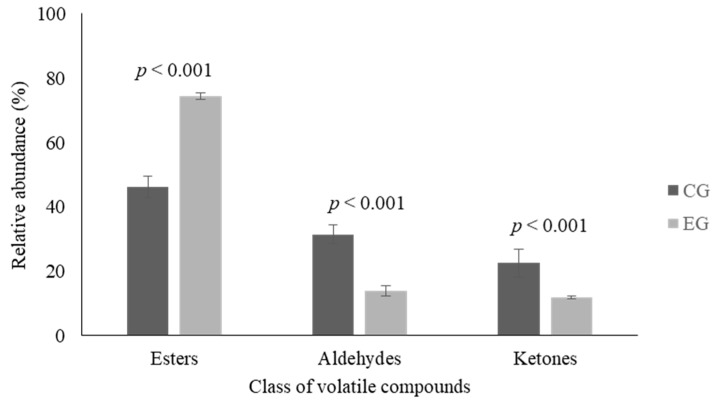
Most representative families of volatile compounds (VOCs) observed in Stracciata samples obtained from control group (CG—dark columns) and experimental group (EG—light columns) after 6 (T6) days. Data are reported as mean percentage ± standard deviation.

In EG samples, as showed in Figure 2, a significant increase (*p* < 0.001) of ester content and a reduction of aldehyde (*p* < 0.001) and ketone (*p* < 0.001) levels were found. As shown in Table 4, several compounds of the ester family were detected in the EG but not in the CG. A significant decrease was detected for octanoic acid, ethyl ester (*p* < 0.05), while no variation between the two groups were observed in 2-butanoic acid ethyl ester and hexanoic acid ethyl ester. On the contrary, both in the CG and the EG Stracciata samples the same aldehydes and ketones were detected, however, no significant differences were observed in relation to the single compounds.

## 4. Discussion

Licorice supplementation did not modify the chemical composition of milk. This finding agrees with other studies that show how the diet supplementation with plants rich in polyphenols and tannins have not effects on milk composition [6,19]. According to what was observed in milk, no significant differences in fat percentage were evidenced in Stracciata obtained from EG milk compared to the CG. However, a lower moisture content was observed in EG Stracciata. Moisture content in cheeses is influenced by many factors as salts, heating of milk, casein, and whey protein. Casein is particularly important in the formation of the structural matrix of the curd that retains fat and moisture. Therefore, the lower moisture in the EG cheeses might have been due to differences in the size and characteristics of the submicelles. Is in fact well known that proteolytic events that occur in dairy products after the cheesemaking may affect casein composition, inducing variations in the ability of proteic fractions to retain water [20].

Significant differences between the two groups were evidenced in the color parameters of Stracciata. Color is often a primary consideration for consumers because it is associated with factors such as freshness, ripeness, desirability, and food safety. Cheese composition, manufacturing procedures, and maturation conditions influence physical properties of cheese such as texture and color [20]. Temperature, technological treatments, and composition parameters such as fat, protein, Ca, and P can influence the physical structure of milk and dairy products and modify the L* color variable of such products. As widely demonstrated, diffusion of light in food matrices, takes place through moisture. In this study the higher L* value was observed in the EG Stracciata samples, which also had a lower moisture content. This apparent contrast suggests that it be discussed in an alternative way. What was observed could be related to a different texture with respect to Stracciata obtained from CG cows. In fact, the dispersion of both casein micelle and fat globules are responsible for the diffusion of incident light and consequently the high L* value. Several studies have highlighted the impact of feed on milk color pigments [21,22]. In addition to what has been reported, variation of b* and a* parameters were also detected in Stracciata cheese and their increase in the EG cheeses might be related to the presence in milk of specific compounds deriving from licorice, such as tannins, carotenoids, and ascorbic acid which are reported to be able to induce variations in color [23].

The results of the present study showed the ability of licorice to influence the FA profile of milk and Stracciata. It is commonly known that FA content in milk is strongly influenced by ruminant diets [24,25,26], and the specific use of licorice root has been reported to be effective in inducing an increase in concentration of palmitic (C16:0) and linoleic (C18:2) acids in ruminants’ diets [27]. Dietary unsaturated FA, mainly C18:2 and C18:3, are extensively metabolized in the rumen [28], starting with the release of free FA (FFA) by the action of lipases. Recent data highlighted the role of plant secondary metabolites, as polyphenols, saponins, and essential oils, to modulate rumen biohydrogenation and, consequently, FA composition of ruminant-derived products [29]. Lipolysis is followed by rumen biohydrogenation, a process consisting of sequential FA isomerization and saturation performed by bacteria to reduce the toxicity of unsaturated lipids for microbial growth [28]. Dietary C18:2 undergoes isomerization by rumen microorganisms in the first step of biohydrogenation which promotes the increase in CLA isomers. The CLA isomers may be reduced to C18:1, t11 and finally to C18:0 in the last step of biohydrogenation. Since the higher amount of C18:2 in EG milk was not correlated to an increase of CLA, C18:1, t11, and C18:0, it is presumable to suppose an inhibitory effect of licorice compounds on the first step of rumen biohydrogenation. Our data disagree with studies of Vasta et al. (2010) [30] and Buccioni et al. (2012) [31] who reported tannins to be able to significantly influence rumen biohydrogenation by improving CLA and C18:1, t11 accumulation. 

Results of investigations to define the role of plant secondary compounds in ruminant nutrition are often contradictory, due to the diversity of active components, dosage, experimental approaches, and ruminant species. Some studies revealed positive modulation of plant compounds, such as tannins, polyphenol oxidase, and oxygenated FA on rumen biohydrogenation [32,33]. Different studies, using either condensed or hydrolysable tannin extracts or tannin rich forages, report a slowdown of initial PUFA metabolism in the rumen [34,35], rather than the specific inhibition of C18:1, t11 saturation that was initially suggested [29]. 

Dosage also represents a relevant issue; Guo et al. (2012) [9] highlighted that the dietary addition of licorice extract in sheep had different effects on rumen fermentation in relation to different levels of licorice supplement and that when more than 4.5% licorice extract was added to the fermentation substrate, both ruminal gas and volatile FA (VFA) production were reduced. A decrease of VFA and acetate and an increase of the levels of propionate and butyrate were observed in Karakul sheep fed with 4.5% of licorice extract [36].

The dietary licorice supplementation decreased the concentration of SFA, particularly of C16:0 which partly derives from the diet and partly from the de novo synthesis by the mammary gland. SFA of up to 16 carbon atoms (short- and medium-chain) are synthesized completely de novo and partially by the mammary gland starting from acetate and beta hydroxy butyrate produced in the rumen [37]. Since no variation in short and medium-chain FA were observed between the milk of the two groups it is possible to hypothesize that the decrease of C16:0 is more related to diet rather than possible effects on mammary gland functionality. The SFA reduction, particularly of C16:0 could improve the nutritional quality of milk and dairy products since their decrease can be beneficial in human health in terms of lowering total and low-density lipoprotein cholesterol. Dietary intake increased the C14:1 and C16:1 levels in milk and this result might be mainly related to the desaturation of C14:0 and C16:0 occurring in the mammary gland by stearoyl coenzyme A desaturase (Δ^9^-desaturase), a finding also supported by the increased values of related ΔI. Stracciata cheese maintained the same differences highlighted in milk. A significant decrease in SFA was observed in EG samples, with a decrease of FA < 12 carbon atoms and an increase of FA > 16 carbon atoms. Contrary to what was observed in milk, dietary supplementation significantly influenced the MUFA and PUFA content in cheese. Further studies would be needed in order to identify the reason why the health functionality of milk is only partially transmitted in the dairy product. The differences with milk might be related to the cheesemaking and to the addition of rennet and starter bacteria.

The evaluation of the oxidative stability of cheese showed interesting differences at T6 and a different trend was observed between the two groups. At T1, in EG Stracciata, the higher MDA levels might be related to higher concentrations of PUFA which are more susceptible to lipoperoxidation. At T6, the MDA values decreased both in CG and in EG Stracciata, however the level of decrease in the EG was two times more than that of the CG. A more marked increase of oxidative stability might be related to secondary metabolites of licorice root, presumably of polyphenolic origin, which may have carried out an antioxidant action [38]. Licorice has received great interest as a natural antioxidant due to its high amount of triterpene, saponins, flavonoids, and phenolic acids. Zang et al. (2015) demonstrated that dietary licorice supplementation in sheep improves in a dose-response manner the antioxidant capacity of meat, by increasing radical scavenging activity and decreasing ROS [8].

The quality of cheese is determined by several factors, such as nutritional features, visual appearances, and flavor, which play a central role in influencing consumer preference. Numerous studies showed that the development of VOCs in cheese is related to milk composition, cheese manufacturing protocol, and microbial activity during ripening. In this study, esters represent the most abundant class of detected VOCs. This is an unexpected finding for a soft cheese, since this class of compounds tends to arise late in the lipolytic process. However these characteristics have been found in similar dairy matrices, as is the case, for instance, of a fresh buffalo milk cheese widely consumed in the Arab world [39]. These compounds are indirectly involved to the metabolism of FFA and their biosynthesis occurs through the activity of lactic acid bacteria, which are common microorganisms in raw milk and cheese. The esterification leads to the formation of esters starting from alcohols and carboxylic acids by the action of esterases [40]. Esters, especially ethyl esters, are responsible for the sweet, floral, and fruity notes of cheeses [41], and their contribution to the flavor of dairy products depends on their concentration; they positively contribute to the overall flavor balance at a low concentration, whereas they are considered off flavors at a high level. With regard to aldehydes and ketones, their formation takes place via lipid oxidation and Maillard reactions. These compounds contribute to the oxidized flavor in dairy products being variously described as being grassy, soapy, metallic, cardboardy, or fishy. The decrease of aldehydes and ketones observed in Stracciata produced by milk obtained from cows fed licorice supplementation could contribute to improving the organoleptic properties of cheese.

## 5. Conclusions

This study highlighted the ability of dietary supplementation with licorice root to influence various parameters of dairy cow products. The reduction of SFA, both in milk and cheese, the increase of MUFA and PUFA, and the modification of volatile profile suggest a positive role of licorice in improving the nutritional and organoleptic properties of cheese. In addition to this, the greater oxidative stability of cheese produced by the milk of cow fed with licorice might improve the shelf-life of dairy product. Further analysis should be performed in order to improve knowledge of the chemical and microbiological mechanisms at the source of these findings.

## Figures and Tables

**Table 1:** Chemical composition of bulk milk obtained from the control group (CG) and experimental group (EG).

	Diet		*p*
	CG	EG	
Casein ^1^	3.12 ± 0.21	3.17 ± 0.27	ns
Lactose ^1^	4.95 ± 0.10	4.86 ± 0.13	ns
Fat ^1^	4.38 ± 0.62	4.64 ± 0.54	ns
Protein ^1^	3.87 ± 0.27	3.98 ± 0.30	ns
Urea, mg 100 mL^−1^	44.01 ± 2.51	49.20 ± 4.57	ns

^1^ Data are expressed as mean percentage ± standard deviation. ns = not significant.

**Table 2:** Chemical and physical evaluations on Stracciata obtained from the control group (CG) and experimental group (EG) after 1 day (T1) from the cheesemaking.

	Diet		*p*
	CG	EG	
***Chemical composition***			
Moisture	69.44 ± 0.73	64.51 ± 0.91	<0.01
Fat ^1^	29.68 ± 4.40	36.90 ± 3.25	ns
***Colour***			
L*	40.38 ± 2.93	44.48 ± 1.83	<0.01
a*	−0.79 ± 0.09	−0.65 ± 0.16	<0.05
b*	1.79 ± 0.32	3.39 ± 0.34	<0.001
YI ^2^	6.42 ± 1.70	10.86 ± 0.68	<0.001
ΔE*ab ^3^	4.40		

^1^ Data are expressed (on a dry matter (DM) basis) as mean percentage ± standard deviation. ns = not significant, L* = lightness, a* = green-red, b* = blue-yellow, ^2^ YI = 142.86 (b*/L*), ^3^ ΔE*ab = [(ΔL*)^2^ + (Δa*)^2^ + (Δb*)^2^]^1/2^.

**Table 3:** Fatty acid composition of bulk milk and Stracciata cheese obtained from the control group (CG) and experimental group (EG).

	Bulk milk			Stracciata		
	Diet		*p*	Diet		*p*
	CG	EG		CG	EG	
C4:0	2.85 ± 0.41	2.75 ± 0.73	ns	3.27 ± 0.46	2.88 ± 0.87	ns
C6:0	2.17 ± 0.30	2.24 ± 0.50	ns	2.83 ± 0.51	2.39 ± 0.69	ns
C8:0	1.43 ± 0.16	1.48 ± 0.28	ns	2.06 ± 0.42	1.59 ± 0.39	ns
C10:0	3.48 ± 0.30	3.50 ± 0.57	ns	5.15 ± 1.08	3.79 ± 0.66	ns
C12:0	3.88 ± 0.22	3.83 ± 0.45	ns	5.04 ± 0.79	4.00 ± 0.33	ns
C14:0	13.41 ± 0.18	13.43 ± 0.58	ns	14.86 ± 1.02	13.30 ± 0.42	ns
C15:0	1.89 ± 0.01	2.12 ± 0.35	ns	1.79 ± 0.02	2.02 ± 0.06	<0.01
C16:0	36.75 ± 0.61	34.31 ± 0.94	<0.01	35.48 ± 1.62	32.49 ± 1.32	ns
C17:0	0.84 ± 0.02	1.10 ± 0.07	<0.001	0.68 ± 0.07	0.94 ± 0.06	<0.01
C18:0	7.65 ± 0.30	6.95 ± 0.63	ns	5.78 ± 0.83	8.26 ± 0.54	<0.05
C20:0	0.19 ± 0.01	0.18 ± 0.04	ns	0.11 ± 0.04	0.22 ± 0.04	<0.05
C22:0	0.13 ± 0.01	0.18 ± 0.07	ns	0.13 ± 0.05	0.15 ± 0.01	ns
SFA	74.66 ± 0.61	72.06 ± 1.51	<0.05	77.04 ± 1.73	71.87 ± 1.03	<0.05
C14:1	0.90 ± 0.01	1.19 ± 0.01	<0.001	0.85 ± 0.07	1.04 ± 0.04	<0.05
C16:1	1.09 ± 0.02	1.25 ± 0.02	<0.001	1.26 ± 0.02	1.04 ± 0.03	<0.001
C18:1 t11	1.09 ± 0.05	0.89 ± 0.04	<0.01	0.67 ± 0.09	1.22 ± 0.04	<0.01
C18:1 c9	15.40 ± 0.50	16.29 ± 1.31	ns	13.37 ± 1.86	16.89 ± 1.14	<0.05
C18:1 c11	0.22 ± 0.01	0.33 ± 0.02	<0.001	0.22 ± 0.04	0.29 ± 0.02	<0.05
MUFA	18.69 ± 0.54	19.94 ± 1.34	ns	16.37 ± 1.92	20.48 ± 1.16	<0.05
CLA	0.63 ± 0.03	0.46 ± 0.03	<0.001	0.54 ± 0.11	0.53 ± 0.04	ns
C18:2	0.97 ± 0.05	1.18 ± 0.06	<0.01	0.98 ± 0.07	1.30 ± 0.10	<0.01
C18:3	0.54 ± 0.02	0.67 ± 0.04	<0.01	0.34 ± 0.25	0.66 ± 0.03	ns
PUFA	2.14 ± 0.08	2.31 ± 0.13	ns	1.86 ± 0.14	2.50 ± 0.16	<0.01
Others	4.53 ± 0.03	5.68 ± 0.06	<0.001	4.59 ± 0.05	5.00 ± 0.55	ns
DI (C14:1)	0.06 ± 0.01	0.08 ± 0.01	<0.001	0.05 ± 0.01	0.07 ± 0.01	<0.001
DI (C16:1)	0.03 ± 0.01	0.04 ± 0.01	<0.001	0.03 ± 0.01	0.03 ± 0.01	<0.01
DI (C18:1)	0.67 ± 0.01	0.64 ± 0.12	ns	0.70 ± 0.01	0.67 ± 0.01	<0.001
DI (CLA)	0.37 ± 0.01	0.34 ± 0.01	<0.01	0.45 ± 0.02	0.30 ± 0.01	<0.001

SFA = saturated Fatty Acid; MUFA = monounsaturated fatty acid; PUFA = polyunsaturated fatty Acid; CLA = conjugated linoleic acid. Desaturation indices (DI): DI C14:1, c9/(C14:0 + C14:1, c9); DI C16:1, c9/(C16:0 + C16:1, c9); DI C18:1, c9/(C18:0 + C18:1, c9); DI CLA/(C18:1, t11 + CLA). Data are expressed as mean percentage of total FA ± standard deviation.

**Table 4:** Volatile profile of Stracciata obtained from the control group (CG) and experimental group (EG) group.

VOC	Stracciata		
	Diet		*p*
	CG	EG	
**Esters**			
2-butanoic acid, ethyl ester	3.24 ± 0.24	0.24 ± 0.06	ns
2-hexanoic acid, methyl ester	nd	5.13 ± 0.65	
ciclopentaundecanoic acid, methyl ester	nd	1.71 ± 0.14	
hexanoic acid, methyl ester	nd	37.39 ± 1.11	
hexanoic acid, ethyl ester	30.59 ± 5.53	11.00 ± 1.03	ns
2-hexanoic acid, ethyl ester	nd	1.91 ± 0.33	
octanoic acid, methyl ester	nd	12.33 ± 1.43	
octanoic acid, ethyl ester	12.29 ± 1.19	3.33 ± 0.30	<0.05
nonanoic acid, 5-methyl, ethyl ester	nd	1.33 ± 0.30	
**Total**	46.12 ± 3.32	74.37 ± 1.07	<0.001
**Aldehydes**			
hexanal	9.23 ± 1.12	4.02 ± 0.51	ns
nonanal	4.43 ± 0.33	1.45 ± 0.06	ns
1-hexanal, 2 ethyl	17.69 ± 1.33	8.35 ± 1.59	ns
**Total**	31.35 ± 2.93	13.82 ± 1.60	<0.001
**Ketones**			
2-heptanone	9.66 ± 0.52	8.02 ± 0.48	ns
2-nonanone	12.88 ± 1.22	3.80 ± 0.43	ns
**Total**	22.54 ± 4.37	11.82 ± 0.46	<0.001

Data are reported as mean percentage of total VOCs ± standard deviation. nd = not detected, ns = not significant.
